# A nomogram to predict in-hospital mortality of gastrointestinal bleeding patients in the intensive care unit

**DOI:** 10.3389/fmed.2023.1204099

**Published:** 2023-09-05

**Authors:** Xueyan Zhang, Jianfang Ni, Hongwei Zhang, Mengyuan Diao

**Affiliations:** ^1^Geriatric Medicine Center, Department of Geriatric Medicine, Zhejiang Provincial People’s Hospital (Affiliated People’s Hospital), Hangzhou Medical College, Hangzhou, Zhejiang, China; ^2^Department of Critical Care Medicine, Affiliated Hangzhou First People’s Hospital, Zhejiang University School of Medicine, Hangzhou, China

**Keywords:** gastrointestinal bleeding, in-hospital mortality, nomogram, intensive care unit, LASSO

## Abstract

**Background:**

Gastrointestinal bleeding (GIB) is a common condition in clinical practice, and predictive models for patients with GIB have been developed. However, assessments of in-hospital mortality due to GIB in the intensive care unit (ICU), especially in critically ill patients, are still lacking. This study was designed to screen out independent predictive factors affecting in-hospital mortality and thus establish a predictive model for clinical use.

**Methods:**

This retrospective study included 1,442 patients with GIB who had been admitted to the ICU. They were selected from the Medical Information Mart for Intensive Care IV (MIMIC-IV) 1.0 database and divided into a training group and a validation group in a ratio of 7:3. The main outcome measure was in-hospital mortality. Least absolute shrinkage and section operator (LASSO) regression was used to screen out independent predictors and create a nomogram.

**Results:**

LASSO regression picked out nine independent predictors: heart rate (HR), activated partial thromboplastin time (aPTT), acute physiology score III (APSIII), sequential organ failure assessment (SOFA), cerebrovascular disease, acute kidney injury (AKI), norepinephrine, vasopressin, and dopamine. Our model proved to have excellent predictive value with regard to in-hospital mortality (the area under the receiver operating characteristic curve was 0.906 and 0.881 in the training and validation groups, respectively), as well as a good outcome on a decision curve analysis to assess net benefit.

**Conclusion:**

Our model effectively predicts in-hospital mortality in patients with GIB, indicating that it may prove to be a valuable tool in future clinical practice.

## Introduction

Gastrointestinal bleeding (GIB) is a common acute and critical disease in clinical practice (1, 2). GIB refers to any bleeding that occurs within the gastrointestinal (GI) tract, which includes the esophagus, stomach, intestine, rectum, and anus. GIB can be divided into upper gastrointestinal bleeding (UGIB) and lower gastrointestinal bleeding (LGIB) according to different bleeding sites. UGIB sites come from the esophagus, stomach, duodenum and near the Treitz ligament; peptic ulcer, esophageal varices and erosive esophagitis are the common causes (3); LGIB is defined as bleeding at the distal end of the Treitz ligament, diverticular bleeding, colitis, and colon polyps are common causes (4, 5); it is often accompanied by hemorrhagic shock, hemodynamic instability, abnormal blood coagulation, organ failure, and even death. Although its incidence has decreased over recent years, the mortality rate is still high. The 30-day mortality rate among patients with upper GIB is 11% (6); it is about 5% among patients with lower GIB (7). In the past, various scores for upper and lower GIB (such as the Glasgow-Blatchford, Rockall, AIMS65, ABC, Oakland, Strategy, NOBLADS, and BLEED score) have been used to assess prognosis (8, 9). These are non-ICU specialty scoring systems commonly used to predict rebleeding, gauge a transfusion rate, or determine ICU admission, and the accuracy of mortality prediction is insufficient [area under the receiver operating characteristic curve (AUROC) <0.8] (10–15).

In the ICU, prolonged mechanical ventilation and abnormal blood coagulation can also cause GIB (16). Critically ill patients are often in a state of stress, and the use of vasoactive drugs reduces gastrointestinal blood flow. Thus, gastrointestinal ischemia, or even necrosis, accompanied by intestinal failure has a poor prognosis. There are few effective assessment criteria for the prognosis of ICU patients with GIB.

Therefore, the main purpose of this study is to find a convenient and practical model to predict the in-hospital mortality of critically ICU patients with GIB to help ICU clinicians to identify and manage high-risk patients.

## Materials and methods

### Source of data

Our data were derived from a publicly available international database, the Medical Information Mart for Intensive Care IV (MIMIC-IV 1.0), a large single-center database covering more than 40,000 critically ill patients admitted to Boston’s Beth Israel Deaconess Medical Center between the years 2008 and 2019. One of the authors of this study (MD) is qualified to extract this database (certificate 1630201).

### Selection of participants

Patients were selected from the MIMIC-IV database based on the following criteria: (1) patients were diagnosed with GIB, whether it was upper or lower GIB, and (2) patients were over 18 years of age. The exclusion criteria were as follows: (1) patients were repeatedly admitted, (2) patients lacked data or were missing important ICU data, (3) patients were not first admitted to the ICU and (4) patients stayed in the ICU for less than 1 day.

### Data extraction

Structure query language (SQL) in PostgreSQL (v14) and Navicat Premium 15 were used to extract clinical study data. We extracted the following: vital signs [age, weight, race, heart rate (HR), respiratory rate, peripheral capillary oxygen saturation (SpO_2_), temperature, and urine output], comorbidities (diabetes, renal disease, chronic pulmonary disease, congestive heart failure, tumor, liver disease, cerebrovascular disease, peripheral vascular disease, peptic ulcer disease), and laboratory parameters [white blood count (WBC), hemoglobin (Hb), hematocrit (HCT), platelets (PLTs), prothrombin time (PT), international normalized ratio (INR), activated partial thromboplastin time (aPTT), blood urea nitrogen (BUN), creatinine (Cr), glucose (Glu), calcium, chloride, sodium, potassium, anion gap (AG), bicarbonate, vasoactive drugs, replacement renal treatment (RRT), ventilator use, sepsis]. Scoring systems [glasgow coma scale (GCS), acute physiology score III (APSIII), Oxford acute severity of illness score (OASIS)]. All of these data were collected during the first 24 h after the patients were admitted to the ICU.

### Outcome

The primary outcome was in-hospital mortality.

### Statistical analysis

The continuous variables in this study were nonnormal distributions; we used the median of the interquartile range (IQR) to express them. The Mann–Whitney test was used for comparison. Categorical variables were expressed as percentages and numbers and were compared using the chi-square test. Patients with GIB were randomly divided into a training group of 1,010 patients and a validation group of 432 patients in the ratio of 7:3. LASSO regression analysis was used for variable shrinking, and variables with regression coefficients equal to zero were eliminated during the contraction process. Then the R language was used to run 10 K cross-validations and to select the best lambda value to obtain the required variables. According to “lambda 1se,” the following variables were obtained: HR, aPTT, SOFA, acute physiology score (APSIII), cerebrovascular disease, AKI, norepinephrine, vasopressin, and dopamine. This was followed by multivariate logistic regression analysis to establish a predictive model and develop a nomogram. STATA (version 17.0) software was used to process the raw data and R Studio (version 4.2.1) for the LASSO regression, logistic regression, and model visualization. *p* < 0.05 was considered statically significant.

## Results

### Patient characteristics

**Figure 1:**
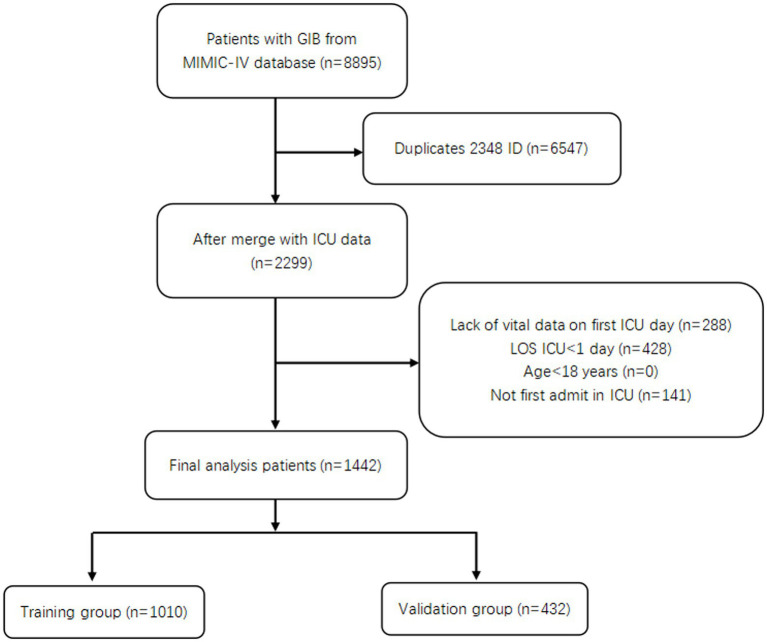
Flowchart of patient selection.

A total of 1,442 patients with GIB admitted to the ICU were screened (Figure 1). Of these, 251 died during hospitalization, resulting in an in-hospital mortality rate of 17.4%. Then 1,010 patients were randomly assigned to a training group and 432 to a validation group, in a ratio of 7:3. Table 1 shows the data on all patients. We can see a total of 745 (51.7%) patients with UGIB, including 530 (36.8%) with non-variceal upper gastrointestinal bleeding (NVUGIB) and 215 (14.9%) with variceal upper gastrointestinal bleeding (VUGIB) 0.252 (17.5%) patients with LGIB, and a total of 445 (30.9%) with bleeding in an unclear site; in terms of in-hospital mortality, NVUGIB, VUGIB, LGIB, and unclear site bleeding were 58 (4.0%), 56 (3.9%), 19 (1.3%), and 118 (8.2%), respectively.

**Table 1:** The baseline of characteristics.

Characteristics	Overall	Training group (*N* = 1,010)	Validation group (*N* = 432)	*p*-value
**Bleeding site**				0.176
VUGIB	215 (14.9)	156 (15.4)	59 (13.7)	
NVUGIB	530 (36.8)	385 (38.1)	145 (33.6)	
LGIB	252 (17.5)	168 (16.6)	84 (19.4)	
Unclear	445 (30.9)	301 (29.8)	144 (33.3)	
**Vital signs**				
Age [median (IQR)]	67.00 [55.00, 79.00]	67.00 [55.00, 79.00]	68.50 [56.00, 80.00]	0.144
Male (%)	877 (60.8)	613 (60.7)	264 (61.1)	0.906
Race (%)				0.413
White	954 (66.2)	680 (67.3)	274 (63.4)	
Black	136 (9.4)	93 (9.2)	43 (10.0)	
Hispanic	50 (3.5)	35 (3.5)	15 (3.5)	
Asian	52 (3.6)	31 (3.1)	21 (4.9)	
Other	250 (17.3)	171 (16.9)	79 (18.3)	
Weight [median (IQR)]	77.90 [66.23, 91.57]	77.90 [67.10, 92.38]	77.75 [64.18, 90.00]	0.233
HR [median (IQR)]	85.40 [74.81, 96.96]	85.63 [75.08, 96.70]	84.91 [73.67, 97.14]	0.711
SBP [median (IQR)]	115.78 [106.05, 128.12]	115.71 [105.79, 128.34]	115.98 [106.51, 127.20]	0.809
DBP [median (IQR)]	61.35 [54.99, 68.48]	61.46 [55.12, 68.80]	61.23 [54.69, 67.89]	0.292
MBP [median (IQR)]	75.52 [69.10, 82.67]	75.52 [69.26, 83.13]	75.53 [68.80, 81.60]	0.643
RR [median (IQR)]	18.46 [16.40, 21.04]	18.50 [16.36, 21.17]	18.32 [16.51, 20.74]	0.6
Temperature [median (IQR)]	36.76 [36.52, 37.02]	36.76 [36.52, 37.03]	36.77 [36.53, 37.02]	0.68
SpO_2_ [median (IQR)]	97.52 [96.19, 98.72]	97.56 [96.19, 98.77]	97.46 [96.16, 98.63]	0.508
Urine output [median (IQR)]	1515.00 [940.00, 2250.00]	1515.00 [964.50, 2275.00]	1515.00 [868.75, 2163.50]	0.446
Glucose [median (IQR)]	127.00 [105.00, 158.30]	127.00 [104.00, 160.70]	126.50 [107.25, 154.05]	0.824
**Comorbidity**				
Congestive heart failure (%)	241 (16.7)	170 (16.8)	71 (16.4)	0.878
Peripheral vascular disease (%)	89 (6.2)	61 (6.0)	28 (6.5)	0.722
Cerebrovascular disease (%)	84 (5.8)	52 (5.1)	32 (7.4)	0.11
Chronic pulmonary disease (%)	221 (15.3)	157 (15.5)	64 (14.8)	0.75
Peptic ulcer disease (%)	244 (16.9)	177 (17.5)	67 (15.5)	0.359
Liver disease (%)	359 (24.9)	265 (26.2)	94 (21.8)	0.073
Diabetes (%)	267 (18.5)	190 (18.8)	77 (17.8)	0.711
Renal disease (%)	213 (14.8)	144 (14.3)	69 (16.0)	0.418
Tumor (%)	133 (9.2)	82 (8.1)	51 (11.8)	0.029
**Laboratory parameters**				
Hematocrit [median (IQR)]	25.40 [22.00, 29.40]	25.50 [22.10, 29.40]	25.00 [21.75, 29.42]	0.331
Hemoglobin [median (IQR)]	8.60 [7.40, 10.10]	8.70 [7.40, 10.10]	8.55 [7.30, 10.10]	0.396
Platelets [median (IQR)]	145.00 [94.00, 214.00]	147.00 [96.25, 213.75]	143.00 [89.75, 217.75]	0.618
WBC [median (IQR)]	8.15 [5.90, 11.60]	8.00 [5.80, 11.50]	8.55 [6.20, 11.90]	0.065
Anion gap [median (IQR)]	16.00 [13.00, 19.00]	16.00 [13.00, 19.00]	15.00 [13.00, 19.00]	0.487
Bicarbonate [median (IQR)]	21.00 [18.00, 24.00]	21.00 [18.00, 24.00]	21.00 [19.00, 24.00]	0.425
BUN [median (IQR)]	31.00 [19.00, 52.00]	30.00 [19.25, 51.00]	32.00 [19.00, 53.00]	0.65
Calcium [median (IQR)]	7.84 [7.40, 8.40]	7.80 [7.30, 8.30]	7.90 [7.40, 8.40]	0.148
Chloride [median (IQR)]	108.00 [104.00, 111.00]	108.00 [104.00, 111.00]	107.00 [104.00, 111.00]	0.178
Creatinine [median (IQR)]	1.10 [0.80, 1.80]	1.10 [0.80, 1.80]	1.10 [0.80, 1.90]	0.703
Sodium [median (IQR)]	141.00 [138.00, 143.00]	141.00 [138.00, 143.00]	141.00 [138.00, 143.00]	0.73
Potassium [median (IQR)]	4.40 [4.00, 5.00]	4.40 [4.00, 5.00]	4.40 [4.10, 5.00]	0.375
INR [median (IQR)]	1.40 [1.20, 1.80]	1.40 [1.20, 1.80]	1.40 [1.20, 1.90]	0.273
PT [median (IQR)]	15.30 [13.20, 19.78]	15.20 [13.20, 19.60]	15.45 [13.40, 20.20]	0.307
aPTT [median (IQR)]	32.50 [27.80, 42.48]	32.45 [27.80, 42.48]	32.75 [27.70, 42.48]	0.7
GCS [median (IQR)]	14.00 [11.00, 15.00]	14.00 [12.00, 15.00]	14.00 [11.00, 15.00]	0.594
SOFA [median (IQR)]	5.00 [2.00, 8.00]	5.00 [2.00, 8.00]	5.00 [2.00, 9.00]	0.939
APSIII [median (IQR)]	47.00 [36.00, 68.00]	47.00 [36.00, 67.00]	48.00 [36.75, 69.00]	0.76
OASIS [median (IQR)]	32.00 [26.00, 40.00]	32.00 [26.00, 40.00]	31.00 [25.00, 39.25]	0.524
Ventilation (%)	431 (29.9)	324 (32.1)	107 (24.8)	0.006
Sepsis (%)	776 (53.8)	559 (55.3)	217 (50.2)	0.084
AKI (%)	315 (21.8)	222 (22.0)	93 (21.5)	0.889
RRT (%)	43 (3.0)	24 (2.4)	19 (4.4)	0.043
Dobutamine (%)	23 (1.6)	15 (1.5)	8 (1.9)	0.648
Dopamine (%)	43 (3.0)	30 (3.0)	13 (3.0)	1
Norepinephrine (%)	334 (23.2)	233 (23.1)	101 (23.4)	0.892
Epinephrine (%)	31 (2.1)	23 (2.3)	8 (1.9)	0.696
Phenylephrine (%)	202 (14.0)	133 (13.2)	69 (16.0)	0.16
Vasopressin (%)	133 (9.2)	88 (8.7)	45 (10.4)	0.321
Neuro-block (%)	44 (3.1)	34 (3.4)	10 (2.3)	0.321
In-hospital death (%)	251 (17.4)	171 (16.9)	80 (18.5)	0.495

### Identification of prognostic factors

**Figure 2:**
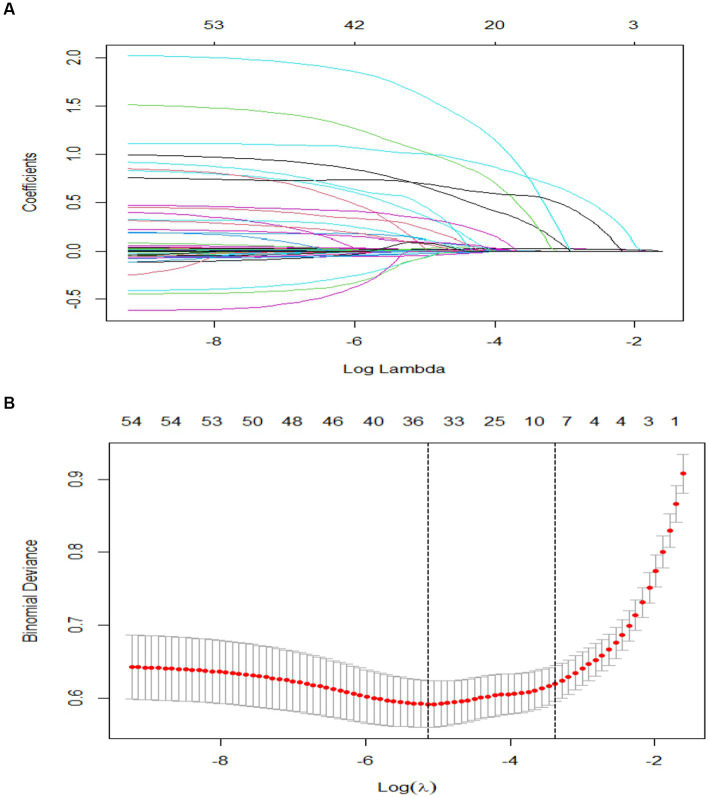
Value selections by LASSO. **(A)** A coefficient profile was plotted against the log (lambda) sequence. **(B)** Nine values with nonzero coefficients were selected by optimal lambda after 10-fold cross-validation. The binomial deviation curve was displayed with log (lambda).

We included a total of 51 relevant variables and selected nine predictors through LASSO regression in the training group (Figure 2). These predictors are HR (OR, 1.02; 95% CI, 1.00–1.04; *p* = 0.001), aPTT (OR, 1.01; 95% CI, 1.00–1.02; *p* = 0.013), APSIII (OR, 1.03; 95% CI, 1.02–1.04; *p* < 0.001), SOFA (OR, 1.02; 95% CI, 0.94–1.10; *p* = 0.616), cerebrovascular disease (OR, 6.98; 95% CI, 3.31–14.69; *p* < 0.001), AKI (OR, 2.06; 95% CI, 1.30–3.29; *p* = 0.002), norepinephrine, vasopressin, and dopamine (OR, 2.97; 95% CI, 1.65–5.34; *p* < 0.001. OR, 1.97; 95% CI, 1.04–3.76, *p* = 0.039. OR, 4.48; 95% CI, 1.62–12.42; *p* = 0.004) (Table 2).

**Table 2:** Multivariate logistic regression analysis of nine independent predictors of in-hospital mortality among patients with GIB.

Values	OR	95% CI	*p*-value
HR	1.02	1.00–1.04	0.001
aPTT	1.01	1.00–1.02	0.013
SOFA	1.02	0.94–1.10	0.616
APSIII	1.03	1.02–1.04	<0.001
Cerebrovascular disease	6.98	3.31–14.69	<0.001
AKI	2.06	1.30–3.29	0.002
Dopamine	4.48	1.62–12.42	0.004
Norepinephrine	2.97	1.65–5.34	<0.001
Vasopressin	1.97	1.04–3.76	0.039

### Development of the prediction model

**Figure 3:**
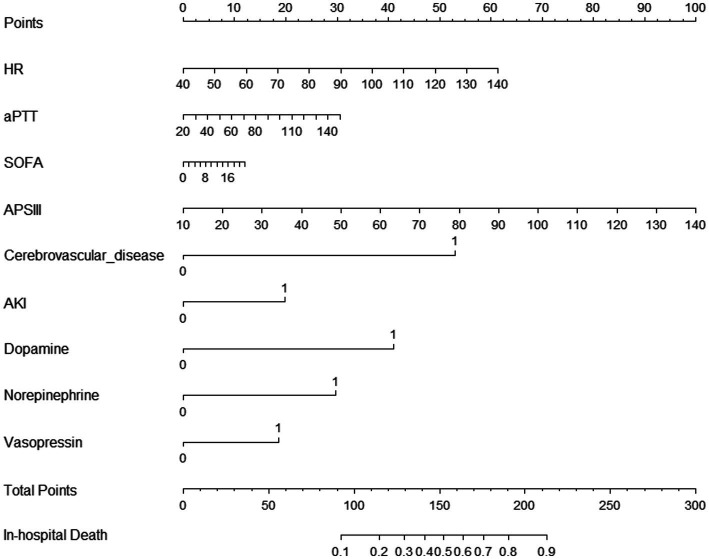
Nomogram model for predicting in-hospital mortality among patients with GIB.

We used the nine predictors to establish a nomogram predicting the probability of in-hospital mortality. Each independent factor in the nomogram was assigned a weighted score, with a maximal score of 240 and a death probability of 0.1–0.9; in this model, the higher the total score on the nine predictors, the higher the probability of in-hospital death (Figure 3).

### Validation of the prediction model

**Figure 4:**
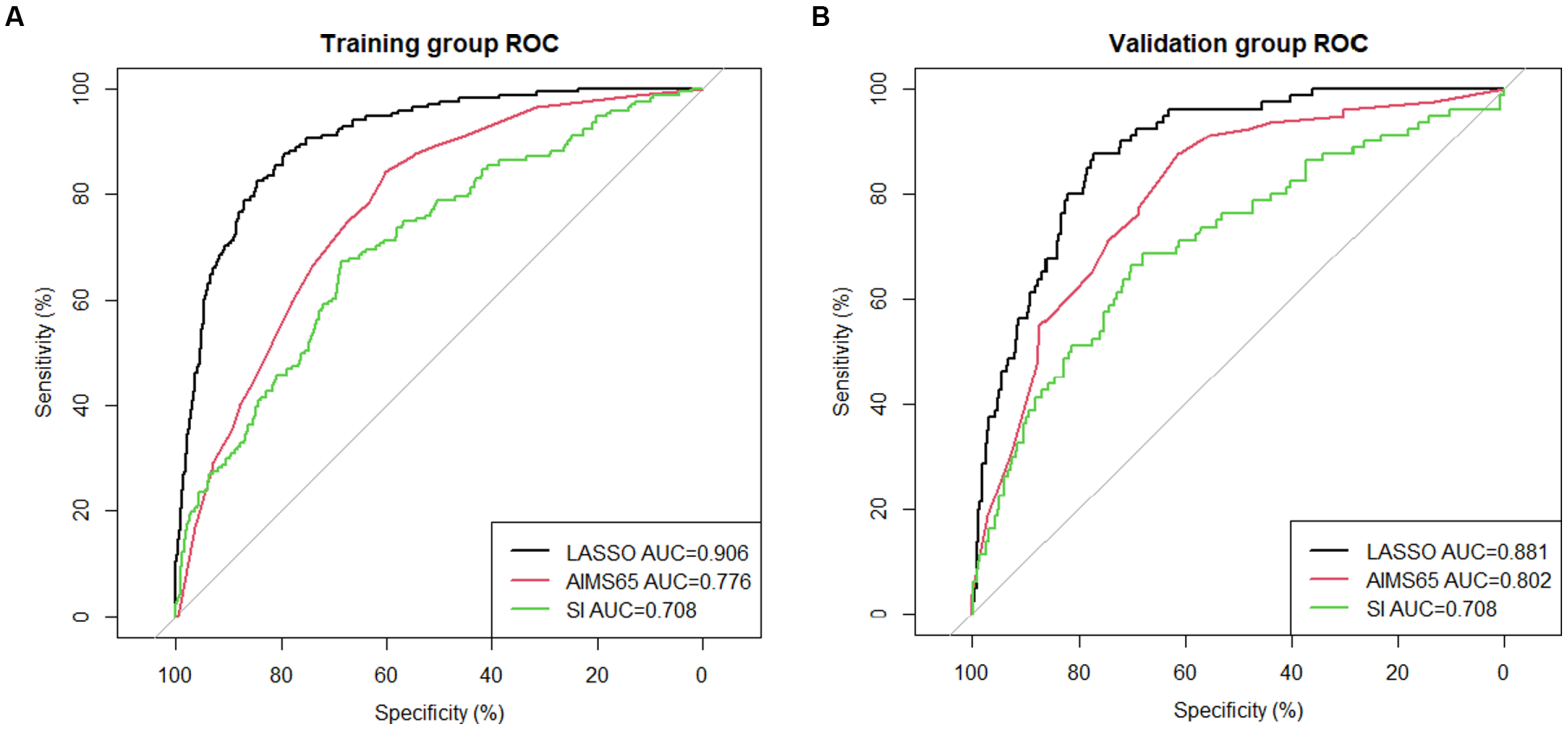
**(A)** ROC curve of the nomogram for predicting in-hospital mortality in the training group; **(B)** ROC curve of the nomogram for predicting in-hospital mortality in the validation group.

**Figure 5:**
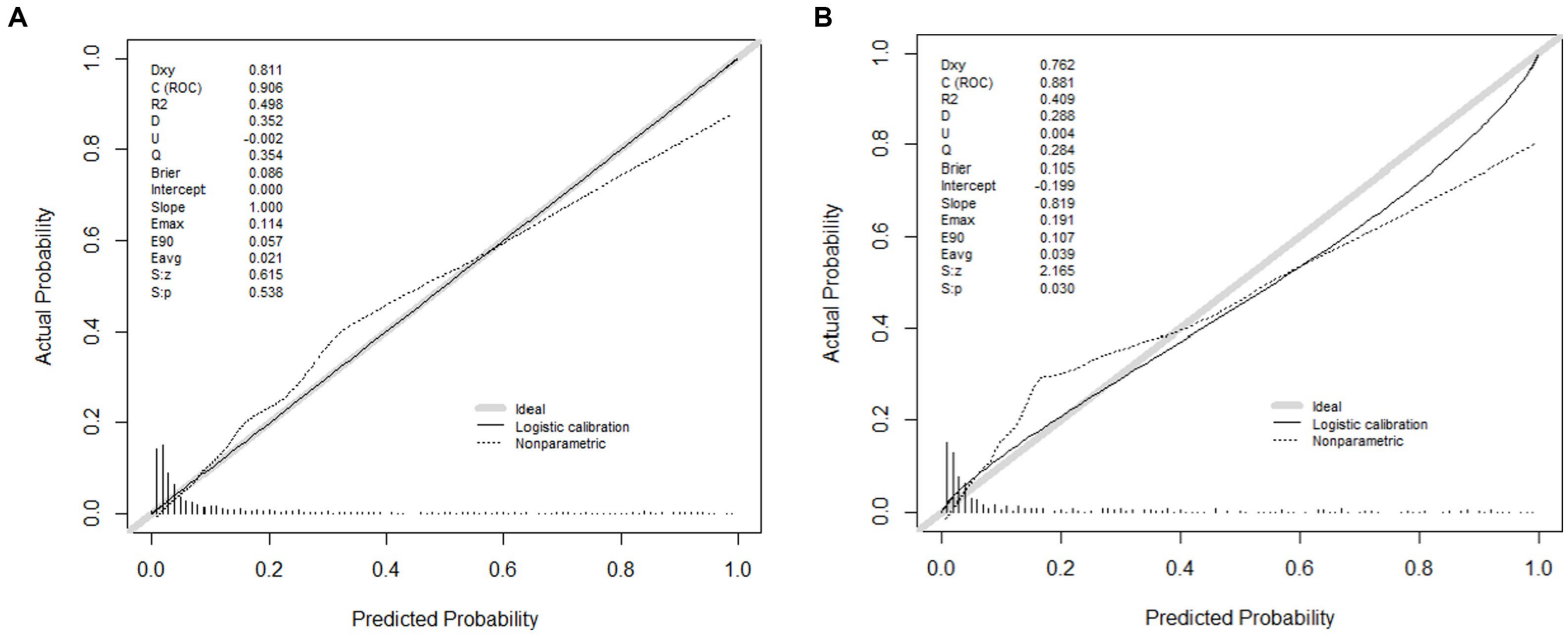
**(A)** Calibration plot of the LASSO model for predicting in-hospital mortality in the training group; **(B)** Calibration plot of the LASSO model for predicting in-hospital mortality in the validation group.

**Figure 6:**
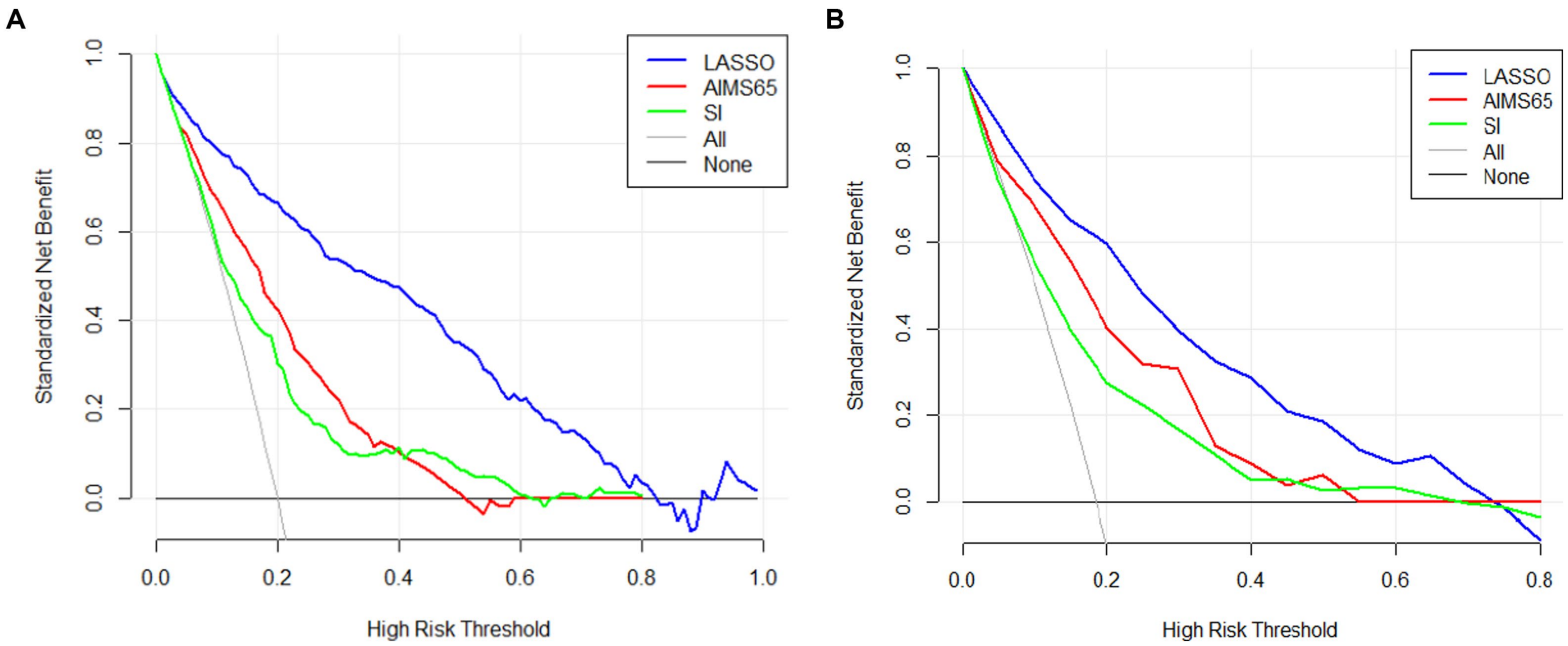
Three difference decision curve analyses (DCAs) of the nomogram models (LASSO, AIMS65, SI) for predicting in-hospital mortality in the training group **(A)** and **(B)** in the validation group.

The area under the nomogram’s ROC curve was 0.906 in the training group and 0.881 in the validation group (Figure 4). The LASSO regression model shows excellent predictive ability; the calibration curve also shows a good fit, as does the Hosmer–Lemeshow test (*p* > 0.05) (training group = 0.35, validation group *p* = 0.10) (Figure 5). We also compared the predictive ability of AIMS65 and SI, in which the AUROC of AIMS65 and SI in the training group was 0.776 and 0.708, respectively. In the validation group, the AUROC of AIMS65 was 0.802, and the AUROC of SI was 0.708. DCA shows that the risk threshold probability in the training group was between 20% and 82%. The risk threshold probability in the validation group was between 20% and 70% (Figure 6). We found it best to use this nomogram to predict the net benefit.

## Discussion

There are many causes of GIB, and most of the existing GIB scores were established by gastroenterology specialists (12, 17), but there are no indicators or scores for predicting the prognosis of critically ill ICU patients with GIB; our study fills this gap. On the basis of our model, which has good discriminative power and net benefits, we produced a user-friendly nomogram that can help clinicians to identify the risk of in-hospital mortality among critically ill patients with GIB in a timely manner.

In our study, patients were categorized into UGIB, LGIB, and unclear site bleeding based on the different sites; UGIB was further categorized into NVUGIB and VUGIB; VUGIB is usually associated with liver disease or portal hypertension, NVUGIB is associated with *Helicobacter pylori* infection, NSAIDs and antiplatelet drug use (18). The incidence of NVUGIB is five times higher than VUGIB; studies in the United Kingdom refer to a 28 days mortality rate of 13.1% for NVUGIB, whereas Japanese and Danish studies have reported a range of 1.1%–11%. Differences in mortality may be related to the methodology and the different populations (19); 30%–50% of patients with varices will experience bleeding, and between 15%–30% of the mortality after bleeding (20). In our research, the in-hospital mortality was (58/530) 10.9% for NVUGIB and (56/215) 26.0% for VUGIB; a guideline on LGIB from the United Kingdom mentions that LGIB in-hospital mortality rate ranges from 3.4%–20%, with an increase in mortality related to whether or not it occurs during hospitalization and the amount of blood transfused after bleeding (21). In addition, there is also a proportion of population that does not have a clear site of bleeding, which tends to be endoscopically negative but with persistent or recurring bleeding, mostly small bowel bleeding. A recent Japanese study recommended computed tomography (CT), small bowel capsule endoscopy (SBCE) and device-assisted enteroscopy examinations for patients with GIB whose site of bleeding is unclear (22). In this study, the in-hospital mortality of patients with LGIB was (19/252) 4.0%, and unclear bleeding site was (118/445) 26.5%, which is much higher than the mortality rate of 5% in the general wards, suggesting us that this type of bleeding deserves more attention in the ICU.

Most of the multiple GIB scores that have existed incorporate age, comorbidities, and important vital signs including blood pressure, heart rate, and consciousness; our predictive model is similar; age is a risk factor for GIB, with older age associated with more comorbidities (23); age was incorporated in Glasgow-Blatchford score, Rockall score, AIMS65, ABC score, PNED score, Oakland score and Sengupta’s study (9). Researches have shown that hospital admissions and hemorrhage for peptic ulcers, increasing in older people, which may be related to the use of anticoagulant drugs and nonsteroidal anti-inflammatory drugs (24, 25); and in older people, the number of LGIB increased which diverticular bleeding is the main reason, the mean age of patients with acute LGIB ranges from 63 to 77 years (26); in our study the median age of GIB patients was around 67–68 years old, the age distribution of the training and validation groups was consistent and not statistically different (67.00 vs. 68.50 years old, *p* = 0.144).

Vital signs are generally recognized as the most important variables in GIB scores; increased heart rate and decreased blood pressure are parameters that reflect the severity of shock. Therefore, the shock index, age shock index and modified shock index were utilized in clinical practice; in Kocaoğlu’s retrospective study, it can be seen that the diagnostic performance of three method is not high in predicting adverse outcomes of GIB (age shock index AUC = 0.711, modified shock index AUC = 0.617, shock index AUC = 0.616) (27); this is consistent with our results (AUC = 0.708). The shock index was similarly inferior to GBS in predicting 30 days mortality in another study of UGIB (AUROC = 0.611 vs. 0.863) (28). Overall, the shock index is not good enough for prognostic diagnosis of patients with GIB.

In 2011, Saltzman developed the AIMS65 score (14). In subsequent studies, it was found that it was superior to the Blatchford score and the endoscopic Rockall score in evaluating the prognosis of patients with upper GIB (29). Not only is AIMS65 discriminatory in the sublinear prediction of UGIB (30, 31), but the literature suggests that AIMS65 are more closely related to mortality than Strate, BLEED, and NOBLADS scores (32). Recent guidelines do not recommend using the AIMS65 score in patients with a low risk of death who do not require hospitalization and endoscopy. This is because the AIMS65 score was designed to be used with high cutoff values to identify patients at high risk for death rather than those at low risk for safe discharge, and about 20% of high-risk patients may be classified as being at low risk (2). In this study, the data value for albumin was missing in more than 20% of the total population; for comparison, we replaced the Charlson Comorbidity Index (CCI) value with the albumin level according to severity; following the previous literature, we replaced patients with a CCI equal to or greater than 5 points with albumin below 30 g/L (33). In our study, the AUC of AIMS65 for predicting GIB mortality was 0.776 and 0.802 in the training and validation groups, respectively.

The CCI is an important scale to classify comorbid conditions which may influence mortality risk, and CCI has high diagnostic power in the prognostic analysis of diseases (34–36). The specific classifications of CCI were detailed in our study, patients with cerebrovascular disease were included as a risk factor for in-hospital mortality from GIB in the predictive model. This may be related to antiplatelet and anticoagulant drugs use (37). This is consistent with NOBLADS and Strate scores. In our study, there were 84 patients with cerebrovascular disease, representing 5.8% of the total number of patients.

APSIII is the most important score used in Acute Physiology and Chronic Health Evaluation (APACHE II); it is useful in the assessment of ICU patients (38). APACHE II is a good tool for predicting hospital mortality in patients with GIB. It can also help to predict adverse outcomes in hospitalized patients undergoing endoscopy (39). APSIII covers 12 physiological indicators—such as general vital signs, inflammatory indicators, and internal environment—which can quantify the risk and degree of multisystem damage in patients in a more comprehensive way. We found APSIII to be a good tool for evaluating in-hospital mortality in ICU patients with GIB (OR, 1.03; 95% CI, 1.02–1.04; *p* < 0.001).

Similar to the APSIII score, the SOFA score includes six major organ systems, namely respiratory, circulatory, coagulation, neurological, renal, and hematological systems, and is often used to indicate the severity of organ dysfunction in ICU patients (40). A higher SOFA score represents more severe organ failure or more organs damage—a condition often accompanied by high mortality. In a study of upper GIB, the qSOFA was selected to be compared with the GBS and Rockall scores, and the qSOFA score was found to be more advantageous in predicting the results of critical care (41). In a risk assessment of patients with NVUGIB, researchers compared the qSOFA and Rockall preendoscopy scores and concluded that the qSOFA score also predicted mortality in patients with NVUGIB (42). In addition, the SOFA score is useful for evaluating GIB and in-hospital mortality among patients with cirrhosis who are undergoing anticoagulation treatment (43, 44). Some researchers have also compared the AIMS65, GBS, Rockall, and ABC scores with APACHE II and SOFA for the prediction of ICU mortality and length of stay. They found that only APACHE II and SOFA had good discriminant values for predicting ICU mortality (AUROC, 0.87; 95% CI, 0.75–0.99; AUROC, 0.71; 95% CI, 0.50–0.93) (45). We also tested the OR value of SOFA in terms of in-hospital mortality in our study (OR, 1.02; 95% CI, 0.94–1.10; *p* = 0.616).

As for AKI, a prospective study showed that the incidence of AKI in the ICU was 57.3%, and increasing severity of AKI was associated with high in-hospital mortality (46). In Cakmak’s study, 102 of 245 patients with UGIB (41%) diagnosed AKI, 32/38 (84.2%) patients who died had combined AKI (47). Patients with renal insufficiency also often have clinical symptoms of GIB because of associated coagulation and vascular disease (48).

Patients with GIB often suffer from hemorrhagic shock owing to severe bleeding. When bleeding is continuous, coagulation factors and platelets in the coagulation system at the bleeding site will be activated, thus promoting thrombus formation (49). Persistent bleeding will lead to a decrease in the number of platelets and coagulation factors, resulting in prolonged coagulation time. Fluid resuscitation will excessively dilute the concentration of coagulation factors, leading to a decline in coagulation function, and finally severe hypothermia, abnormal coagulation, and acidosis will greatly increase the mortality rate (50).

The use of vasoactive drugs is an indicator of the severity of hemodynamics. The need for vasoactive drugs in patients with GIB often represents a situation where the circulation has been compromised or is facing collapse. Norepinephrine, vasopressin, and dopamine were also significantly independent factors in patients with GIB. According to the current guidelines, whether it is upper or lower GIB, fluid resuscitation and blood transfusion are recommended first. Vasoactive drugs increase blood pressure and maintain organ perfusion. Norepinephrine and dopamine are often recommended in clinical practice (51). Norepinephrine and dopamine cause peripheral vasoconstriction and increase cardiac output by stimulating α1 and β1 receptors (52), whereas vasopressin increases blood volume by inducing V2 aquaporin to increase water reabsorption. It can also interact with V1a receptors to contract vascular smooth muscle cells, thus increasing blood pressure (53).

### Limitations

The limitations of this study are reflected in the following. First, this was a single-center retrospective study, so that there is an inevitable risk of bias affecting the test results. Second, the data studied did not include albumin, myocardial enzymes, or other biochemistry elements. In the statistical analysis of the raw data, any variable with missing values greater than 20% was excluded from the analysis. Third, the fact that we relied on the MIMIC database to split the total number of patients into a training set and a validation set without any external data validation may affect the applicability of the model.

## Conclusion

We found that HR, aPTT, APSIII, SOFA, cerebrovascular disease, AKI, norepinephrine, vasopressin, and dopamine were independent predictors of in-hospital mortality in patients with GIB. We therefore developed a nomogram that can accurately predict in-hospital mortality in patients admitted to the ICU with GIB. We have also provided a convenient tool for clinicians, especially those working in the ICU, for identifying patients with poor prognoses who may require further diagnosis and treatment.

## Data availability statement

The original contributions presented in the study are included in the article/supplementary material, further inquiries can be directed to the corresponding author.

## Ethics statement

The MIMIC-IV database was established by the Massachusetts Institute of Technology (Cambridge, MA) and Beth Israel Deaconess Medical Center (Boston, MA), and consent was obtained for the original data collection. Therefore, the ethical approval statement and the need for informed consent were waived for the studies on this database.

## Author contributions

JN conceived and designed this study. MD extracted data from MIMIIC database. XZ is responsible for processing data and completing the manuscript. HZ participated in the writing of the manuscript and the correction of the final version. All authors contributed to the article and approved the submitted version.

## Conflict of interest

The authors declare that the research was conducted in the absence of any commercial or financial relationships that could be construed as a potential conflict of interest.

## Publisher’s note

All claims expressed in this article are solely those of the authors and do not necessarily represent those of their affiliated organizations, or those of the publisher, the editors and the reviewers. Any product that may be evaluated in this article, or claim that may be made by its manufacturer, is not guaranteed or endorsed by the publisher.
